# Spacer Designs for Improved Hydrodynamics and Filtration Efficiency in Sea Water Reverse Osmosis

**DOI:** 10.3390/membranes15010032

**Published:** 2025-01-16

**Authors:** Sarah Kerdi, Adnan Qamar, Henry J. Tanudjaja, Noreddine Ghaffour

**Affiliations:** Environmental Science and Engineering Program, Division of Biological and Environmental Science and Engineering (BESE), King Abdullah University of Science and Technology (KAUST), Thuwal 23955-6900, Saudi Arabia; adnan.qamar@kaust.edu.sa (A.Q.); noreddine.ghaffour@kaust.edu.sa (N.G.)

**Keywords:** feed spacer, reverse osmosis, direct numerical simulations, concentration polarization, hydrodynamics

## Abstract

Reverse osmosis (RO) filtration performance is heavily influenced by the design of the feed spacer. Spacer design impacts hydrodynamic patterns within the system, affecting water production and concentration polarization. Two spacer designs, namely pillar (P) and standard (S), were investigated to improve the performance of a commercially available spacer design (C) in the RO process. Two approaches were employed to evaluate spacer performance. First, direct numerical simulation (DNS) was utilized to fundamentally understand the hydrodynamics generated by each spacer design. Second, laboratory RO experiments were conducted to confirm the simulation results. The P and S spacers induced higher flow velocity and vorticity than the C spacer, as confirmed by simulations and experiments. Reduced dead zones were also demonstrated using P and S spacers. However, the standard spacer design exhibited a clear advantage in promoting more efficient mixing within the filtration channels. This enhanced mixing substantially reduced salt concentration at the membrane surface, improving the filtration performance. In agreement with the permeation velocity computation, the S spacer achieved the highest improvement (13%) in both flux yield and specific flux relative to the C spacer. This finding confirms the S spacer’s ability to enhance RO performance while reducing energy consumption.

## 1. Introduction

A harsh competition for water resources has emerged between industries, agriculture, and public users. Consequently, the increased demand for freshwater and the continuing population growth have led to water shortages worldwide [1]. Securing natural freshwater supplies is imperative for sustaining current and future water demands. Desalination technologies such as alternative resources to produce clean water have been developed over the last few decades [2]. The most widely employed desalination technology is reverse osmosis (RO) due to its propensity to provide a high quantity and quality of freshwater with modest operating costs [1,3]. RO is a pressure-driven filtration process whereby all the smallest contaminants and dissolved constituents can be rejected over a semi-permeable membrane by applying hydraulic pressure, which results in highly purified water production [2,4]. Spiral wound membrane modules (SWMs) are the predominant commercial configuration for the RO process [5]. Industries favor them due to their easy scale-up operation, high permeation, and fouling control possibility. The suitability of the feed spacer design in SWMs is an important consideration for RO process improvement.

Besides the inter-membrane spacing role, the feed spacer in SWMs plays a crucial role in determining the hydrodynamic conditions in filtration channels [6]. It acts as a fluid mixer, helping to promote unsteadiness/turbulence in the feed flow over the membrane surface [7]. This reduces the fouling (organic, inorganic, colloidal, and biofouling) on the RO membrane, mitigating concentration polarization, scaling, and crystallization phenomena, which are the bottleneck factors for RO membrane performance [8,9]. Moreover, an enhancement in mass transfer occurs along with a reduction in pressure loss, thereby producing more clean water at a lower cost [5,10]. Optimizing spacer characteristics for an enhanced RO process remains challenging. The engineering of an optimal feed design is linked to its potential to appropriately manage the fluid flow over the membrane surface to improve the filtration performance and mitigate the fouling/concentration polarization by controlling the hydrodynamics in the feed channels [11]; hence, this must be studied further.

The commercial spacer design has a net-type shape of non-woven filaments creating a narrow-filled channel with low porosity, which increases the channel pressure drop and energy requirements. Furthermore, dead zones are frequently localized near the spacer filaments’ intersections, which favors fouling accumulation with the filtration evolvement at these stagnant membrane areas [12,13]. Therefore, improvement of the commercial design is needed to overcome its fundamental limitations. Research efforts have been focused on altering its geometric parameters such as the spacer orientation, internal angle, thickness, mesh size, etc. [14,15,16,17]. However, the amendment in hydrodynamics could not adequately eliminate the limitations related to the asymmetric shear stress/velocity distribution that arises from the non-uniform filament cross-sections [13]. Hence, exploring appropriate feed spacer designs is urgently required to eradicate the complicated hydrodynamics nature of the commercial design and facilitate its transition to an unsteady state.

Recently, many advanced 3D-printed spacers have been developed such as triply periodic minimal surfaces, turbo-spacers, honeycomb spacers, etc. [9,18,19,20]. Created by our research group, a symmetric standard spacer characterized by even and cylindrical filament cross-sections was significantly able to overtake the non-uniform shear stress distribution concern promoted by the commercial design [21]. Based on standard spacer design, the tradeoff balance was shifted towards enhanced mass transfer with respect to reduced energy costs. Therefore, a modification in microstructure features of the standard design was carried out along the spacer filaments (creation of perforations, helices, etc.) and at filament intersections (pillar-type nodes) to improve the hydrodynamics and simultaneously minimize the pressure drop [13,21]. Indeed, the induced hydrodynamic alteration helped to effectively boost the localized velocity filtration channel, resulting in higher water production and lower biomass accumulation in ultrafiltration (UF) processes. The pillar spacer design was particularly distinguished by its ability to increase the channel porosity (i.e., lower the pressure drop) and mitigate the biofouling deposition [13]. Moreover, pillar-type nodes created additional vortex shading bodies behind the filaments’ intersections, intensifying fluid perturbation with the shortness of dead zones. More recently, the pillar-type spacer revealed further its potential to enhance the promoted shear stress (i.e., reduced concentration polarization) by simply raising the relative pillar size in its microstructural design [22].

The performances of standard and pillar spacer designs revealed promising anti-biofouling tendencies and improved water production for the UF process, where bacterial-active feed solution is typically utilized [21]. Thus, the hydrodynamic alteration induced by these designs was evaluated in terms of biomass accumulation. However, their efficiency in reducing the concentration polarization phenomenon (i.e., mostly encountered in the RO process) has so far been ignored. The local velocity and shear stress variations in filtration channels differentially impact biofouling and concentration polarization phenomena. Although high shear stress areas favor the attachment of bacteria and their steady growth in hydrodynamic nature [23], they are desirable to minimize the concentration polarization [24,25]. Therefore, the engineering of optimal spacer design is intimately connected to the desired process application. Encouraged by their effectiveness in improving UF performance, standard and pillar spacers (named S and P spacers, respectively) were assessed for an enhanced RO process in the present manuscript. The hydrodynamics induced by these spacer designs were numerically simulated for the RO process using direct numerical simulation (DNS). Many hydrodynamic parameters describing the fluid flow behaviors within the filtration channel such as the local velocity distribution, vorticity magnitude, and salt concentration were investigated utilizing S and P spacers and compared to their patterns of commercial spacers (named C spacer). Furthermore, the permeation velocity (i.e., which reflects the mass transfer that occurred through the RO membrane) was further examined. Later, the feed spacers were 3D-printed and experimentally tested in a lab-scale RO system to validate the modeling results through permeate flux and specific flux monitoring.

## 2. Materials and Methods

### 2.1. Numerical Approach

The hydrodynamic patterns in filtration channels were assessed for the different tested spacers using the DNS approach. The Reynolds number was calculated as *ρU_o_ H/μ*, where *ρ* is the density, *U_o_* is the average inlet velocity, *H* is the channel thickness, and *μ* is the dynamic viscosity. Turbulent models were not used, as the DNS method effectively solved the full Navier–Stokes equations with appropriate meshing. However, in cases where the flow could transition to turbulence due to geometric factors, careful meshing techniques were employed to accurately capture any turbulent scales in both spatial and transient domains. The simulations were carried out for both complete spacer cells and half spacer cells. The governing Navier–Stokes equations are as follows:

(1) $$\nabla \cdot \left(\vec{u}\right)=0$$

(2) $$\frac{\partial \vec{u}}{\partial t}+\nabla \cdot \left(\vec{u}\;\vec{u}\right)=-\frac{1}{\rho }\nabla P+\frac{µ}{\rho }\nabla^{2}\;\vec{u}+\frac{1}{\rho }f$$

$$\frac{\partial c}{\partial t}+\nabla \cdot \left(\vec{u}\;c\right)=\nabla \cdot \left[D\;\nabla \;c\right]$$

where *t* represents time, $\vec{u}$ is the velocity field vector (*u* as the x-component, *v* as the y-component of velocities), *P* is the pressure, *f* is the external force, *c* is the salt concentration, *D* is the salt diffusivity, and ∇ is the three-dimensional spatial gradient operator. The osmotic pressure of the incoming feed at various spatial locations is computed using the Van’t Hoff formula:

(3) $$\pi_{f}=iMRT\;$$

where *i* is the Van’t Hoff factor, *M* is the molar concentration of dilution (mol/L), *R* is the ideal gas constant (8.314 J mol^−1^ K^−1^), and *T* is the temperature (K). *T* was fixed to 294 K corresponding to the experiments for all simulations.

At the start of the RO filtration process, the membrane is not instantly fouled and at this permeate rejection rate, *q* (m/s) during the early ages of filtration can be assumed to follow Darcy’s law:

(4) $$q=-\frac{K\;∆p}{\mu \;L}\;$$

where *K* is the permeability of the membrane (m^2^), ∆p is the transmembrane pressure (Pa) and *L* is the thickness of the RO membrane (m). The transmembrane pressure is the combination of system-applied pressure and local osmotic pressure difference given by the following equation:

(5) $$∆p={∆p}_{ap}-iMRT-iMRT\;\left(1-R_{c}\right)\;$$

where ${∆p}_{ap}$ is the applied mechanical pressure from the pump side (operating pressure), and *R_c_* is the rejection of the RO membrane. It is important to note here that this formulation does not take into account fouling as the simulations are intended to evaluate the design of the RO feed spacer at the early stages of filtration.

In the simulations, the fluid was assumed to have Newtonian properties, and the membrane surface was considered to have permeation. At the membrane surface, a slip wall boundary condition was imposed (*u* = 0, *v* = *q*) at all spatial discretized faces of the membrane. At the inlet of the computational domain, uniform flow conditions are prescribed (*u* = *U_o_*, *v* = 0, and *c* = *c_o_*). Periodic boundary conditions are applied in the spanwise direction to account for the symmetry and replication of the spacer cells. Finally, at the membrane surface, the salt concentration is updated by integrating the boundary layer film model [26]:

(6) $$c_{m}=c_{o}\left(1-R_{c}\right)+\left[c_{c(i,j)}-c_{o}(1-R_{c})\right]e^{\frac{\left|q\right|\;dr}{D}}\;$$

where *c_m_* is the local salt concentration at each time step, $c_{c(i,j)}$ is the cell-centered concentration value computed by solving Equation (3), and *dr* is the spatial distance between the cell center and the cell face on the membrane surface.

To solve the governing equations and boundary conditions, we utilized the commercial solver ANSYS Fluent 2022 R1 within a finite-volume framework [27]. The computational domain was divided into 12.6 million control volumes, employing second-order formulations for spatial and temporal discretization. The convective term in the pressure formulation was discretized using the QUICK (Quadratic Upstream Interpolation for Convective Kinematics) method [28]. The interaction between pressure and velocity was managed using the PISO (Pressure Implicit with Split Operator) algorithm. Due to the computational complexity, the simulations were carried out using 1024 cores on an Intel Haswell Processor with 2 CPU sockets per node, 16 cores per CPU, operating at 2.3 GHz, and 128 GB of memory per node, provided by the KAUST Supercomputing facility (SHAHEEN II) [29].

### 2.2. Geometric Characteristics of Feed Spacers

S and P spacers were in-house prototyped utilizing Computer-Aided Design (CAD) on SolidWorks software (Dassault Systemes SolidWorks Corporation, Version 2018) for experimental performance evaluation. Then, they were manufactured via UV polymerization of acrylate monomers (liquid resin, BV-007, MiiCraft Inc., Hsinchu City, Taiwan) using 3D printing technology (Model 125, Version 3.4.5, MiiCraft Inc., Hsinchu City, Taiwan) with a layer thickness of 25 µm. The C spacer was commercially provided and extracted from the SWM module (Dow Filmtec, SW30HRLE-400 Seawater RO Element, Dow Chemical, Minnesota, MN, USA).

**Figure 1:**
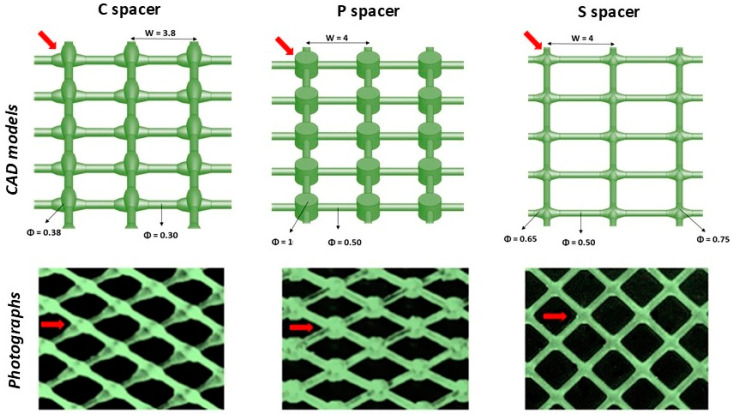
CAD with geometric dimensions and 3D-printed photographs of the commercial (C), pillar (P), and standard (S) feed spacers. The red arrows represent the direction of the fluid flow. All the dimensions on the CAD designs are in mm. W is the mesh length and Φ is the diameter of the spacer filaments and filament intersections.

The geometric parameters of all tested spacers are summarized in Figure 1 along with the CAD and spacer photography. A thickness of 0.78 mm for the 3D-printed S and P spacers was selected, matching that of spacers commonly used in commercial RO SWMs.

### 2.3. Experimental Protocol for RO Tests

**Figure 2:**
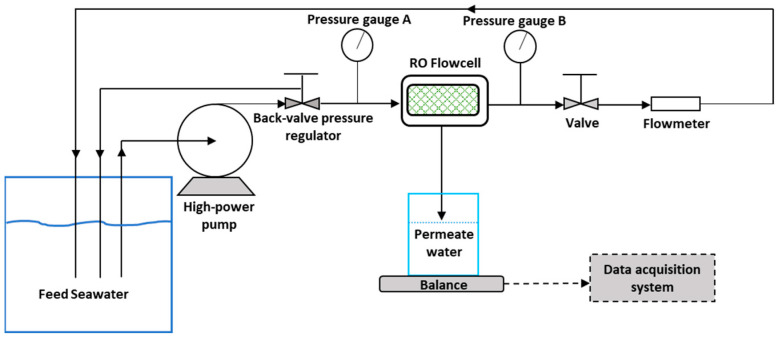
Schematics of the cross-flow RO lab-scale system.

Polyamide flat-sheet membrane coupons were extracted from a commercial RO SWM module (DOW FILMTEC™ SW30HRLE–400 Seawater RO Element, Dow Chemical, Minnesota, MN, USA) for all RO experiments. The membrane and feed spacer coupons were enclosed in a custom-made flow cell with a channel height of 0.78 mm. The filtration active area of the membrane coupons was 7.7 cm × 2.6 cm. The schematic of the RO lab-scale setup is depicted in Figure 2. A high-power pump (Hydra-cell pump, Model M03-S, Wanner International, Hampshire, UK) was used to feed the seawater to the flow cell unit. Natural Red Sea water, which was directly delivered to the Water Desalination and Reuse Center (WDRC) laboratory through a pipeline [30], was used as a feed solution for all tests. Two digital pressure gauges (Digi-Sense, Model EW-68920-20, Cole Parmer, Vernon Hills, IL, USA) were located at the input and output of the flow cell to monitor the applied hydraulic pressure of 60 ± 2 bar. A flowmeter (Model n° RMB-SSV, Dwyer Instruments, London, UK) was placed at the flow cell outlet to set the volumetric flow rate (Q) at 350 mL/min (equivalent to an inlet velocity of U0 = 0.27 m/s, Reynolds number Re~218) in the feed channel. The feed pressure and flow rate were controlled using two built-in mechanical valves. The produced permeate water was regularly collected in a tank installed on a digital balance (Model MS3002S, Mettler Toledo, Columbus, OH, USA) to automatically record every 5 min the produced water weight via a data acquisition system (National Instruments, LabView software, Version 2016) throughout RO operation.

## 3. Results and Discussion

The numerical spacer design optimization offers a reactive approach to amend the fluid flow behavior over the membrane surface, thereby establishing the local hydrodynamics inside filtration channels [31]. Accordingly, tracking the fluid behavior at an elemental level using DNS simulations helps to elucidate the different hydrodynamic parameters induced by spacer design modification. Therefore, the variation in local velocity magnitudes was investigated for all tested spacer designs in order to verify the unsteady/turbulent state and fluid mixing conditions in filtration channels. Subsequently, the permeation velocity and salt concentration distributions in the RO membrane were further computed to evaluate the spacer design’s performance in terms of permeate flux enhancement and concentration polarization reduction. The validation of the DNS results was experimentally achieved by measuring the permeate flux produced in the RO process along with specific flux that allowed us to estimate the energy required for the different spacer designs.

### 3.1. Localized Flow Field Velocity Distribution

**Figure 3:**
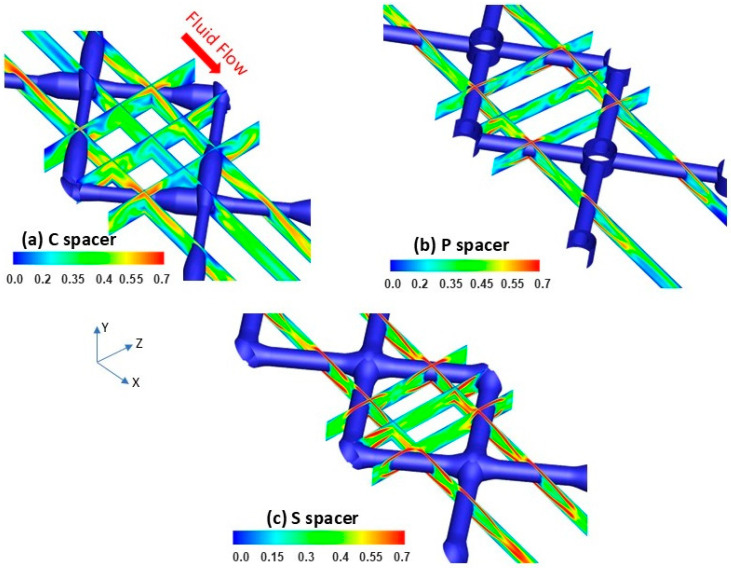
DNS spatial velocity magnitude (m/s) contours on X-Y and Y-Z planes taken at various locations of filtration channels for (**a**) commercial (C), (**b**) pillar (P), and (**c**) standard S spacers.

Figure 3 shows the local velocity magnitude variation for different locations of the various spacer-filled channels computed at an inlet flow velocity of U0 = 0.27 m/s, which is subsequently applied in RO experiments. As seen in Figure 3a, an asymmetric flow field was noticed for the C spacer reflecting its non-woven design. The localized velocity was uneven around the cross-sectional C spacer filaments, where its magnitude appeared more pronounced (~0.5–0.6 m/s) on the face hosting the incoming fluid flow. Behind the filament intersections, dead zones with minimum local velocity (~0–0.2 m/s) were identified. These stagnant regions are known to be vulnerable to high particulate settlement and fouling deposition [16,32]. Unsteady hydrodynamics prevailed at the middle of C spacer cells showing oscillated flow velocity within the range of ~0–0.4 m/s.

In contrast, a uniform flow velocity distribution was manifested within P and S spacer-filled channels, which was attributed to their symmetric designs (Figure 3b,c). Unlike the C spacer, the down and up fluid streams were evenly segregated under P and S spacer filaments and increased in velocity magnitude to achieve local mass and momentum conservation at the constricted regions [33]. The velocity magnitude (~0.7 m/s) under the filaments of P and S spacers was higher than that of the C spacer due to the reduced clearance space created between these spacer filaments and the membrane surface [34]. Furthermore, additional eddies were performed behind P and S filament intersections, predicting reduced dead zones compared to the commercial design. Although the local velocity at the middle area of P spacer cells exhibited an unsteady behavior oscillating in the range of ~0–0.4 m/s, a steady hydrodynamics nature was manifested for the S spacer with higher up-limit velocity magnitude reaching ~0.5 m/s. Contrary to C and P spacers, the greater velocity magnitude was found to invade the entire central area of S spacer cells. Consequently, a higher fluid shear stress and lower salt deposition were foreseen utilizing the standard spacer design.

### 3.2. Spatial Flow Vorticity Distribution

**Figure 4:**
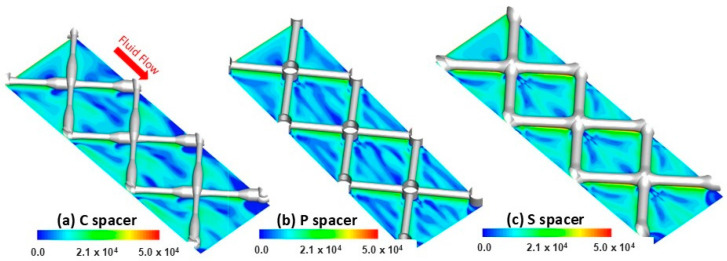
DNS spatial vorticity magnitude (s^−1^) contours at the plane near the RO membrane walls for (**a**) commercial (C), (**b**) pillar (P), and (**c**) standard (S) spacers.

The vorticity is a parameter that defines the local fluid rotation and forecasts the velocity gradient in filtration channels [35]. An important flow vorticity promotes additional fluid mixing, which improves the mass transfer and mitigates the concentration polarization [36]. Figure 4 illustrates the spatial local vorticity distribution extracted from the computational domain at the plane near the membrane walls for the different spacers. The highest vorticity magnitude was identified under the filaments for all spacers, indicating enhancement of fluid mixing effects at these regions [37]. Following the velocity, the vorticity magnitude was uneven along the C spacer filaments due to the varied filament cross-sections (i.e., non-uniform clearance) (Figure 4a). However, it was homogeneous under the filaments of P and S spacers having with uniform cross-sections (i.e., uniform clearance) (Figure 4b,c). Extensive low-vorticity regions (dark blue zones with nil intensity) were observed within C and P spacer cells (Figure 4a,b), with slightly higher intensity for commercial design. However, the curl flow rotation (i.e., fluid mixing) was greater (~5.9 × 10^3^ s^−1^ within S spacer cells (Figure 4c)) in harmony with the highest localized velocity observed in Figure 3c.

Based on the present numerical results, it has been predicted that the standard design has the highest fluid vorticity. Thus, the standard spacer has the potential to generate a more efficient mixing effect relative to the other spacers resulting in enhanced filtration performance and reduced concentration polarization. Furthermore, it is noteworthy to emphasize that the localized velocity and vorticity intensities displayed similar spatial distribution patterns in filtration channels for all spacer configurations. Beyond the localized velocity/shear stress, the computation of the flow vorticity further revealed its potential as a valuable numerical tool for understanding hydrodynamics and anticipating fouling on the RO membrane surface.

### 3.3. Localized Salt Concentration Field Simulation

**Figure 5:**
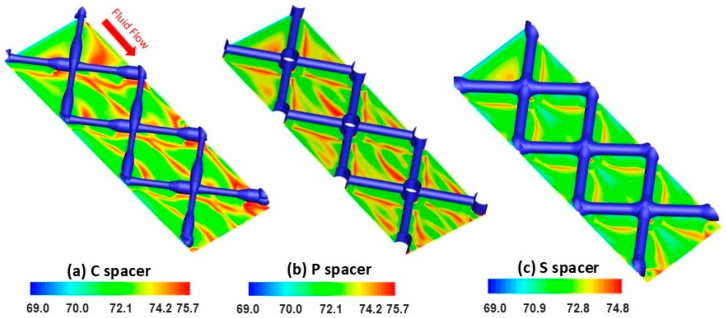
DNS calculation of the salt concentration (mol/L) contours in the vicinity of RO membrane walls for (**a**) commercial (C), (**b**) pillar (P), and (**c**) standard (S) spacers.

The localized salt deposition on the RO membrane surface was simulated to investigate the impact of spacer design on the concentration polarization phenomenon. The formation of the concentration polarization layer increases the osmotic effects due to the accumulation of salts on the membrane surface resulting in a lowering of the mass transfer in the boundary layer and declining in the permeate flux production [38]. Figure 5 exhibited the local salt concentration contours calculated in the different spacer-filled channels.

In the streamwise direction, the salt accumulation followed a repeated pattern along the membrane surface regardless of the spacer designs. For the C spacer, the regions under the thin-filament side trapped more salts than the thick-filament side due to the variation in velocity extents (Figure 5a). The higher velocity magnitude, which was promoted under the thick-filament side (i.e., due to the lower clearance), swiped the salts away, alleviating the salt concentration in these regions [39,40]. Under P and S spacer filaments, the salt concentration was found homogenous with lower intensity (Figure 5b,c), which is attributed to the higher flow motion on the membrane surface induced by the reduced clearance created by these spacer designs compared to the commercial design. For all spacers, the salt was remarkably trapped behind the filament intersections due to the weakness of flow mixing at these stagnant zones [41]. Within the spacer cells, the salt concentration field appeared to reincarnate the flow vorticity map distribution. Interestingly, the membrane areas with nil vorticity magnitude (dark blue zones, as seen in Figure 4) reflected high salt deposition tendency (red zones, as seen in Figure 5) for all spacer cases. The standard design, characterized by maximal local vorticity/velocity at the center of spacer cells, significantly reduced the salt concentration magnitude. In harmony with the vorticity distribution, the low-magnitude salt concentration area was quasi-uniformly dragged into the whole S spacer cell areas resulting in mitigated concentration polarization relative to the other designs. Consequently, an improvement in filtration performance by increasing water flux was anticipated using the standard spacer design and is demonstrated in the subsequent sections.

**Figure 6:**
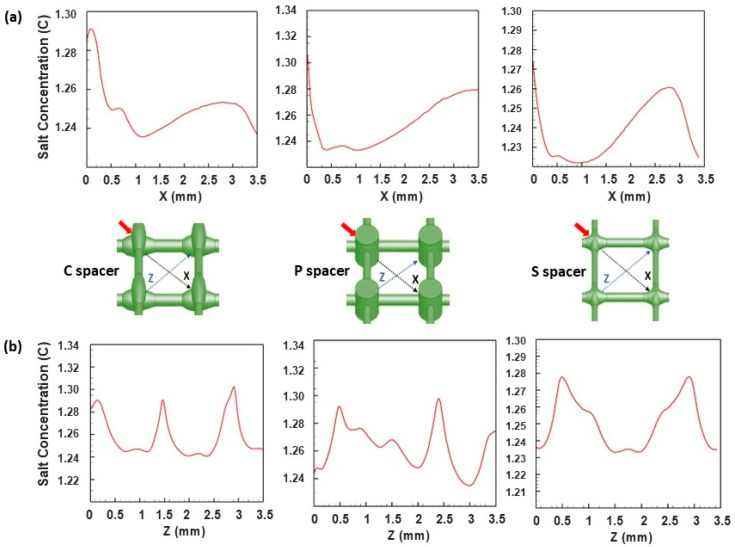
Localized elementary salt concentration (mol/L) profiles calculated inside the computational domain of commercial (C), pillar (P), and standard (S) spacers’ spacer-filled channels in the streamwise (**a**) and spanwise (**b**) flow direction. The red arrows represent the flow direction.

Salt concentration profiles were further extracted from the computational field at spatial locations of the different spacer-filled channels to thoroughly investigate the changes in salt concentration along (X direction) and perpendicular (Z direction) to the flow direction (Figure 6). In the streamwise direction (Figure 6a), the highest salt concentration peaks were estimated in the range of C~1.26–1.3 mol/L and appeared behind the filament intersections, where the stagnant flow was located due to the attachment of spacers to the membrane surface. At these regions, the lowest value associated with a narrow peak of the local salt concentration (C~1.26 mol/L) was noticed for the standard design, indicating dead zones’ alleviation achieved by this spacer. Moving toward the center of the spacer cells, the P spacer had the greatest salt accumulation with a broad peak reaching a value of C~1.28 mol/L. In the spanwise direction (Figure 6b), the salt concentration profiles revealed a significant oscillation in the deposited salts’ magnitude in cases of C and P spacers, affirming the unsteady hydrodynamic nature previously demonstrated in localized velocity distribution (Figure 3). However, a lower fluctuation in the salt concentration profile was observed for the standard design in a way that aligns with the quasi-uniform salt intensity distribution and the steady hydrodynamic nature detected within the cells of this spacer.

### 3.4. Localized Mass Transfer Distribution

**Figure 7:**
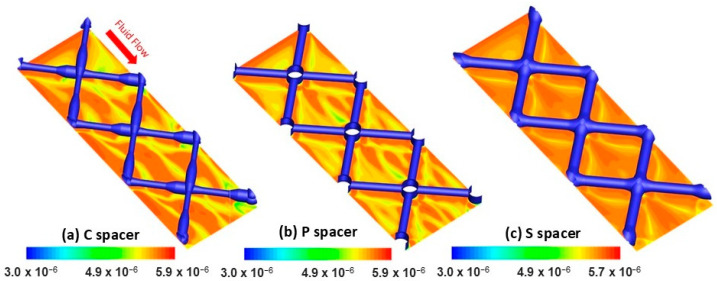
DNS calculation of the permeation velocity contours near RO membrane walls for (**a**) commercial (C), (**b**) pillar (P), and (**c**) standard (S) spacers.

The mass transfer distribution was further investigated via computation of the permeation velocity, which helped to estimate the fluid capability to pass through the RO membrane. This numerical analysis aids in figuring out the impact of spacer design on filtration performance. As seen in Figure 7, the permeation velocity contour plots computed in the vicinity of RO membranes varied significantly depending on the spacer design. Alternated low and high permeation velocity intensities were identified for C and P spacers (Figure 7a,b), whereas a greater magnitude quasi-uniformly covered the entire cells of the S spacer (Figure 7c). The map of local permeation velocity distribution on the membrane surface was well correlated to its counterpart of salt concentration/vorticity regardless of the spacer design. Hence, the higher permeation velocity regions (i.e., the higher passage of permeate flux through the membrane) in spacer cells coincided with those with higher local vorticity and lower salt accumulation tendency. Likewise, it is noteworthy to highlight that the membrane areas under the spacer filaments that demonstrate a high local velocity consistently revealed a high-flux permeation velocity magnitude. Therefore, the local water flux production tends to increase under the spacer filaments, where a low potential of salt deposition was demonstrated, as shown in Figure 5.

In conclusion, the higher promotion of localized velocity/vorticity along with lower salt concentration was achieved using the standard spacer. Thus, based on the numerical insights, this spacer design outperforms the pillar and commercial designs in improving the flux production and mitigating the concentration polarization in the RO process.

### 3.5. Experimental Validation of DNS Simulations in RO Process

The computation by DNS allowed a quick and effective assessment of the different spacer designs in improving the hydrodynamics and exploring fundamentally the effects in terms of concentration polarization and mass transfer distribution on the RO membrane surface. The numerical approach and grid independence applied in the present study are thoroughly detailed and validated in previous research work by utilizing a commercial spacer [36]. On the other hand, RO tests were conducted using a lab-scale system to experimentally evaluate the filtration and energy performance of the various spacer designs.

**Figure 8:**
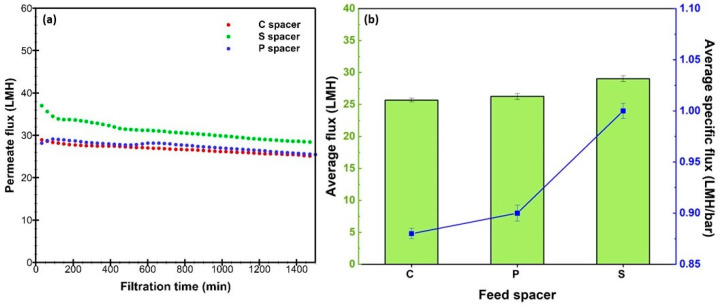
Experimental performance of commercial (C), pillar (P), and standard (S) spacers tested in lab-scale RO system: (**a**) permeate flux profile during filtration and (**b**) average flux with specific flux. The presented average values are calculated under steady-state conditions of the RO process.

Operating under the same conditions, the permeate flux was monitored over a 24 h RO process and the specific flux was determined for each spacer design (Figure 8). The S spacer has a higher flux than C and P spacers throughout the whole filtration, which confirms the beneficial effects that are observed from the simulation. C and P spacers produced a comparable permeate flux with an average value of 26.0 LMH when a flux steady-state circumstance was achieved. However, the S spacer achieved a higher flux, averaging approximately ~29.1 LMH. Interestingly, the standard design exhibited its potential to improve flux production with a 13% enhancement percentage relative to the commercial design.

Simultaneously, the energy performance was further examined through the specific flux calculation for all RO tests. The specific flux parameter quantifies the amount of water flux produced per unit of net applied pressure, which is around 30 bar (applied pressure of 60 bar minus Red Sea osmotic pressure of around 30 bar) [42], thereby revealing the energy efficiency of the different spacer designs. While the C and P designs exhibited a comparable value of specific flux (~0.88–0.90 LMH/bar), the S design resulted in a higher value (~1.0 LMH/bar), consequently indicating that an energy-beneficial filtration process occurred by integrating this spacer into the RO module.

As performed by DNSs, the standard spacer revealed the highest local velocity magnitude due to its reduced clearance space between the spacer and membrane surface, supported by a localized velocity/vorticity that quasi-uniformly covered most of the membrane area due to the symmetrical design. These help in decreasing dead zone areas and increasing the permeation rate. Furthermore, this spacer design has the potential to promote substantial fluid mixing by helping to reduce the salt accumulation on the membrane surface, which prevents the concentration polarization effect, reduces fouling tendency, and ultimately reduces the energy requirements for a long-term RO operation. These numerical outcomes were confirmed in practice and expressed experimentally by a significant enhancement in water flux production and specific flux in the filtration channel equipped by the S spacer, thus demonstrating a more efficient RO filtration and reduced energy costs.

The next steps are to test the fouling mitigation with this improved spacer design and scale-up study, as well as a cost–benefit analysis. Fouling testing will show the comprehensive benefits of the S spacer design, as it has been tested only for flux enhancements. Fouling in the form of organic, inorganic, or biofouling is commonly found in the seawater reverse osmosis process [8]; hence, it is critical that the spacer’s ability to mitigate this phenomenon is tested in the future.

Scaling up can be difficult to achieve. As the tested spacers are 3D-printed in a small size for lab-scale testing, the resolution, accuracy, structural strength and print quality can be effectively controlled, but it will be challenging to maintain the same parameters on a bigger scale, especially with a more complex spacer design. In addition, it is more costly than the conventional extrusion method [43]. Other factors are the manufacturing speed and capacity of the 3D printer to print an industrial size that is used in the spiral wound membrane module. Currently, 3D-printed membranes are not yet ready to compete with the conventional extrusion method due to these factors [43], which limits their application to a bigger scale. However, as technology and knowledge evolve over time, 3D-printed spacers will be able to fulfill their potential in the near future.

## 4. Conclusions

In the present study, the effectiveness of pillar and standard spacer designs in enhancing RO technology was investigated and compared to the available commercial spacers. The hydrodynamics induced by these spacers were comprehensively analyzed using the direct numerical simulation approach and confirmed experimentally using RO tests. The following has been demonstrated:

- The vorticity magnitude distribution map on the membrane surface strongly correlated with those of velocity and salt concentration, with areas of high vorticity coinciding with areas of high velocity and low salt concentration.
- More efficient fluid mixing was achieved by the standard spacer translated by a greater velocity/vorticity magnitude in the middle of spacer cells, resulting in enhanced permeation velocity and mitigated concentration polarization.
- Compared to the commercial spacer, the standard spacer increased permeate and specific flux by 13% in RO tests, demonstrating a more efficient and energy-reduced process when integrated into RO SWM elements.

## Data Availability

The raw data supporting the conclusions of this article will be made available by the authors on request.
